# Interrogating the CD27:CD70 axis in αCD40-dependent control of pancreatic adenocarcinoma

**DOI:** 10.3389/fcell.2023.1173686

**Published:** 2023-04-12

**Authors:** Awndre Gamache, Claire Conarroe, Sara Adair, Todd Bauer, Frederic Padilla, Timothy N. J. Bullock

**Affiliations:** ^1^ Department of Pathology, School of Medicine, University of Virginia, Charlottesville, VA, United States; ^2^ Beirne B. Carter Center for Immunology Research, University of Virginia, Charlottesville, VA, United States; ^3^ Department of Surgery, University of Virginia, Charlottesville, VA, United States; ^4^ Focused Ultrasound Foundation, Charlottesville, VA, United States; ^5^ Department of Radiology, School of Medicine, University of Virginia, Charlottesville, VA, United States

**Keywords:** CD40, CD27, CD70, TIL activation, PDAC, KPC, CPI therapy

## Abstract

Immune checkpoint blockade immunotherapy has radically changed patient outcomes in multiple cancer types. Pancreatic cancer is one of the notable exceptions, being protected from immunotherapy by a variety of mechanisms, including the presence of a dense stroma and immunosuppressive myeloid cells. Previous studies have demonstrated that CD40 stimulation can remodel the tumor microenvironment in a manner that promotes effector immune cell responses and can cooperate with immune checkpoint inhibition for durable tumor control mediated by T cells. Here we confirm the capability of this combination therapy to dramatically, and durably, control pancreatic cancer growth in an orthotopic model and that the immune memory to this cancer is primarily a function of CD4^+^ T cells. We extend this understanding by demonstrating that recruitment of recently primed T cells from the draining lymph nodes is not necessary for the observed control, suggesting that the pre-existing intra-tumoral cells respond to the combination therapy. Further, we find that the efficacy of CD40 stimulation is not dependent upon CD70, which is commonly induced on dendritic cells in response to CD40 agonism. Finally, we find that directly targeting the receptor for CD70, CD27, in combination with the TLR3 agonist polyIC, provides some protection despite failing to increase the frequency of interferon gamma-secreting T cells.

## Introduction

Pancreatic ductal adenocarcinoma (PDAC) is notoriously resistant towards immune checkpoint inhibitor therapy (CPI) (Royal et al., 2010; Bian and Almhanna, 2021). Several features have been implicated in the lack of response to CPI, including a dense fibrous stroma that limits immune cell and molecule penetration; the presence of immunosuppressive myeloid cells; and a low mutational burden limiting the availability of antigenic epitopes available to effector T cells (Balli et al., 2017). Therefore, it is imperative to explore alternative approaches to activate and enhance the immune system’s response in this context.

CD40 is a tumor necrosis factor receptor superfamily member that is expressed on dendritic cells (DC), macrophages, monocytes, and B cells(Bullock, 2022). CD40 can be engaged by CD40 ligand (CD40L), which is expressed by activated CD4^+^ T cells and NKT cells and leads to cellular activation. This process drives B cell survival and contributes to class switching, and is the critical activation step for licensing DC to initiate CD8^+^ T cell responses to cross-presented antigens (Bennett et al., 1998; Ridge et al., 1998; Schoenberger et al., 1998; Pérez-Melgosa et al., 1999). Agonist αCD40 monoclonal antibodies (mAbs) have been used to simulate CD40L engagement *in vivo* and circumvent the need for CD4^+^ T cell help (Rowley and Al-Shamkhani, 2004; Bullock and Yagita, 2005; French et al., 2007; McWilliams et al., 2010; Vonderheide and Glennie, 2013). Moreover, CD40 has been combined with various therapies, including TLR agonists, chemotherapy, radiotherapy, and checkpoint inhibitors, to increase the performance of cancer vaccines and enable immunological control of tumors (Vonderheide, 2020; Burrack et al., 2021).

Despite earlier studies that have shown that CD40 agonism is extremely potent against PDAC, the mechanistic basis for these anti-tumor capabilities is poorly understood. On the one hand, CD40 stimulation can drive a T cell-independent activation of macrophages that is sufficient to curtail tumor growth (Beatty et al., 2011). On the other, subsequent studies have implicated a role for the CD40-mediated activation of conventional DC (cDC) leading to T cell-dependent control of experimental PDAC (Winograd et al., 2015; Byrne and Vonderheide, 2016; Rech et al., 2018; Morrison et al., 2020; Byrne et al., 2021). The basis by which CD40-mediated activation of DC drives T cell responses in the context of PDAC is being elucidated. Various immunologically-relevant molecules are produced by cDC after the agonism of CD40, and blocking innate components, such as type-1 interferons, modestly limits the therapeutic activity of CD40 stimulation (Morrison et al., 2020). Pertaining to this, the CD70:CD27 axis has been shown to be a critical component in bridging CD40-mediated activation of DC and adaptive immunity in vaccination and cancer (Rowley and Al-Shamkhani, 2004; Bullock and Yagita, 2005; Feau et al., 2012). Stimulation of CD27 drives the expression of Eomesodermin, and critical cytokine receptors associated with T cell differentiation and survival (Dong et al., 2012). Further, stimulation of CD27 in combination with the IFNαβ receptor results in a synergistic expansion of CD8^+^ T cells and is associated with the induction of the effector T cell transcription factor, T-bet. Studies in other systems have implicated a role for CD70 in the anti-tumor activity of CD40-stimulation (French et al., 2007; Oba et al., 2020). Thus, we hypothesized that the effectiveness of CD40 stimulation at limiting the growth of PDAC is mediated, in part, by the induction of CD70 on cDC.

Here, we employed a syngeneic murine LSL-Kras^G12D/+^, LSL-Trp^53R172H/+^, Pdx1^Cre^ (KPC) cell-implantation model to investigate the underlying mechanism for αCD40-induced tumor control (Byrne and Vonderheide, 2016; Long et al., 2016). We validate previous findings that αCD40 and CPI treatment results in tumor regressions and immunological memory(Morrison et al., 2020). We find that the intratumoral reservoir of T cells present at the time of CD40 activation is sufficient to mediate tumor control, without recruitment of newly primed T cells from the tumor-draining lymph node. However, surprisingly we find that the activity of αCD40 is independent of CD70, but can in part be replicated by the provision of CD27 stimulation in combination with the TLR agonist polyIC (pIC), which potently induces the expression of interferons.

## Materials and methods

### Mice

6–8 week-old C57BL/6J mice were purchased from Jackson Laboratories. Mice were housed in a controlled environment that was free of specific pathogens, and their treatment was conducted in compliance with the Animal Care and Use Committee’s guidelines at the University of Virginia (Charlottesville, Virginia).

### Tumor implantation models

Two LSL-Kras^G12D/+^, LSL-Trp^53R172H/+^, Pdx1^Cre^ (KPC) cell lines were used as indicated. KPC4662 was kindly provided by the Vonderheide laboratory (Byrne and Vonderheide, 2016). KPC7940b was procured from the Beatty laboratory (Long et al., 2016). For subcutaneous (s.c.) implantations, 250k tumor cells were implanted in 100ul of 1:1 matrigel (Corning) and DPBS (Gibco). Tumor volume estimates were based off length and height measurements *via* calipers as described (Sápi et al., 2015). Mice were humanely euthanized to collect tumors at specified time points, once they had grown to their maximum permitted size, or if wet ulcers had developed.

For the orthotopic experiments, we implanted 500k KPC4662 cells in a 25 µL suspension of 1:1 matrigel and DPBS or RPMI (as indicated), using the same procedure as previously described (Stokes et al., 2011). Tumor measurements were taken *via* ultrasonography and volume was calculated as described (Faustino-Rocha et al., 2013).

For rechallenge experiments, mice were implanted with tumor cells on the opposing rear flank for the initial rechallenge, and on either fore flanks for tertiary and quaternary implantations.

Blocking antibodies: αPD1 (200ug; RMP1-14), αCTLA4 (200ug; 9H10), αCD70 (600ug; FR70), agonist antibodies: αCD40-agonist (100ug; FGK45), or αCD27-agonist (100ug; AT124) (BioXcell, Ichor Biosciences, or Celldex), and depletion antibodies: αCD8 (250 μg; 2.43) and αCD4 (250 μg; GK1.5) were all administered intraperitoneally (i.p.) as indicated.

Brefeldin A (BFA) (250ug; Selleckchem), was injected i.p. 6 h prior to euthanasia. pIC (100ug) were administered i.p. as indicated. Fingolimod (FTY720; Sigma) was administered as previously described (Stevens and Bullock, 2021).

### Flow cytometry

Flow cytometry was performed using an Aurora Northern Lights (Cytek) or Attune™ NxT Acoustic Focusing cytometers (Thermo Fisher). Data were collected using Spectroflo or Attune NxT software and analyzed using FlowJo (version 10.8). After titrating for optimal resolution, fluorescent mAbs from BD Biosciences, BioLegend, Invitrogen, and Phitonex were used to stain for CD45 (30-F11), MHCII (2G9), CD3 (145-2C11), CD127 (SB/199), CD44 (IM7), TCRγδ (GL3), CD11c (HL3), CD27 (LG 3A10), CD8α (5H10), Ly6G (1A8), XCR1 (ZET), F4/80 (T45-2342), CD11b (M1/70), CD4 (GK1.5), CD172a (P84), CD19 (6D5), Ly49G2 (4D11), NKp46 (AF700), NK1.1 (PK136), EOMES (Dan11mag), FOXP3 (FJK-16s), IFN-γ (XMG1.2). LIVE/DEAD fixable dye from Thermo Fisher Scientific was also used to evaluate viability of cells.

Freshly harvested tumors were minced, homogenized, and underwent lympholyte-M (Cedarlane) gradient centrifugation for immune cell isolation. Samples were viability and surface stained prior to fixation with the Cytofix/Cytoperm, or FOXP3 fixation, kit (BD) for intracellular staining.

### Statistical analysis

GraphPad Prism (GraphPad Software, Inc.) was employed to compute all statistical analyses. Mantel-Cox test was employed for survival analysis. Two-way ANOVA or a mixed effects model was used to determine significance of difference in tumor growth among multiple treatment arms. The Holm-Šídák multiple comparison, or Brown-Forsythe and Welch ANOVA tests, were used to define significance among groups. Symbols (*, **, ***, ****) are used to denote *p* values < 0.05, 0.01, 0.001, or 0.0001, respectively.

## Results

Checkpoint inhibition and CD40 diminish discreet T_reg_ cell populations in the tumor microenvironment (TME)

**FIGURE 1:**
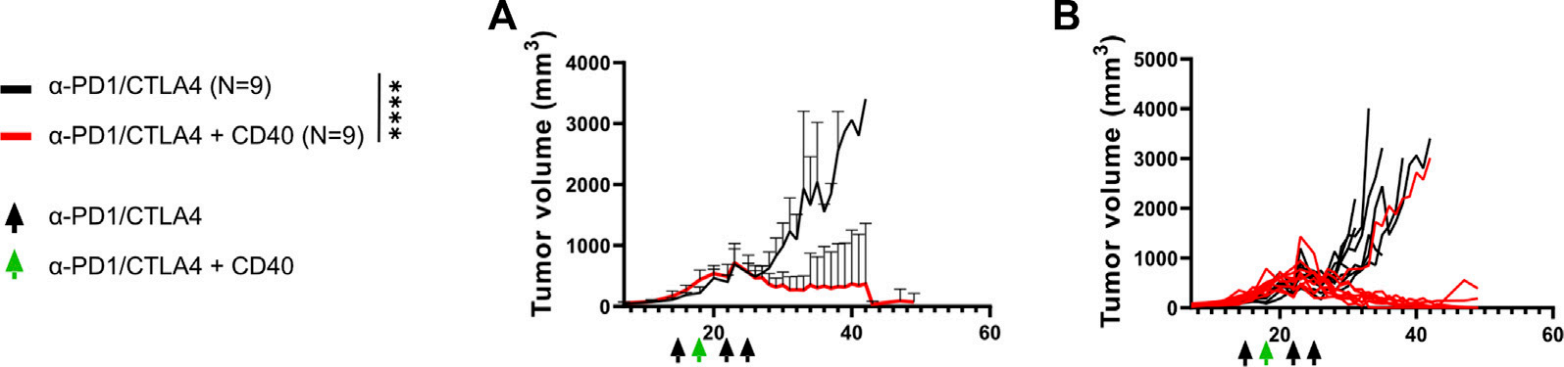
CPI40 treatment promotes robust PDAC tumor regression. 250k KPC-4662 tumor cells were implanted s.c. into C57BL/6mice. **(A)** Average and **(B)** individual tumor growth curves over time. Black arrows denote administration of αPD1 (200 ug) and αCTLA4 (200 ug), the green arrow denotes administration of the former plus αCD40 (100 ug). Mice were treated on days 15, 18, 22, and 25. *N* = 9 per group. For average curves, maximal tumor volume was repeated in this experiment for mice that reached endpoint before day 35. Mixed-effects analysis was performed, *p* < 0.0001 across time and treatment. This experiment was performed once.

We initially evaluated the effectiveness of PD1 and CTLA4 checkpoint inhibitors and CD40 (CPI40) treatment in suppressing KPC4662 tumors in a C57BL/6J mouse model. The treatment with CPI40 produced a noticeable reduction in tumor growth, both in orthotopically and subcutaneously implanted tumors (Supplementary Figure S1; Figures 1A, B). These data suggest that CPI40 treatment can lead to robust tumor control independent of additional treatment interventions and is effective against well-established tumors.

**FIGURE 2:**
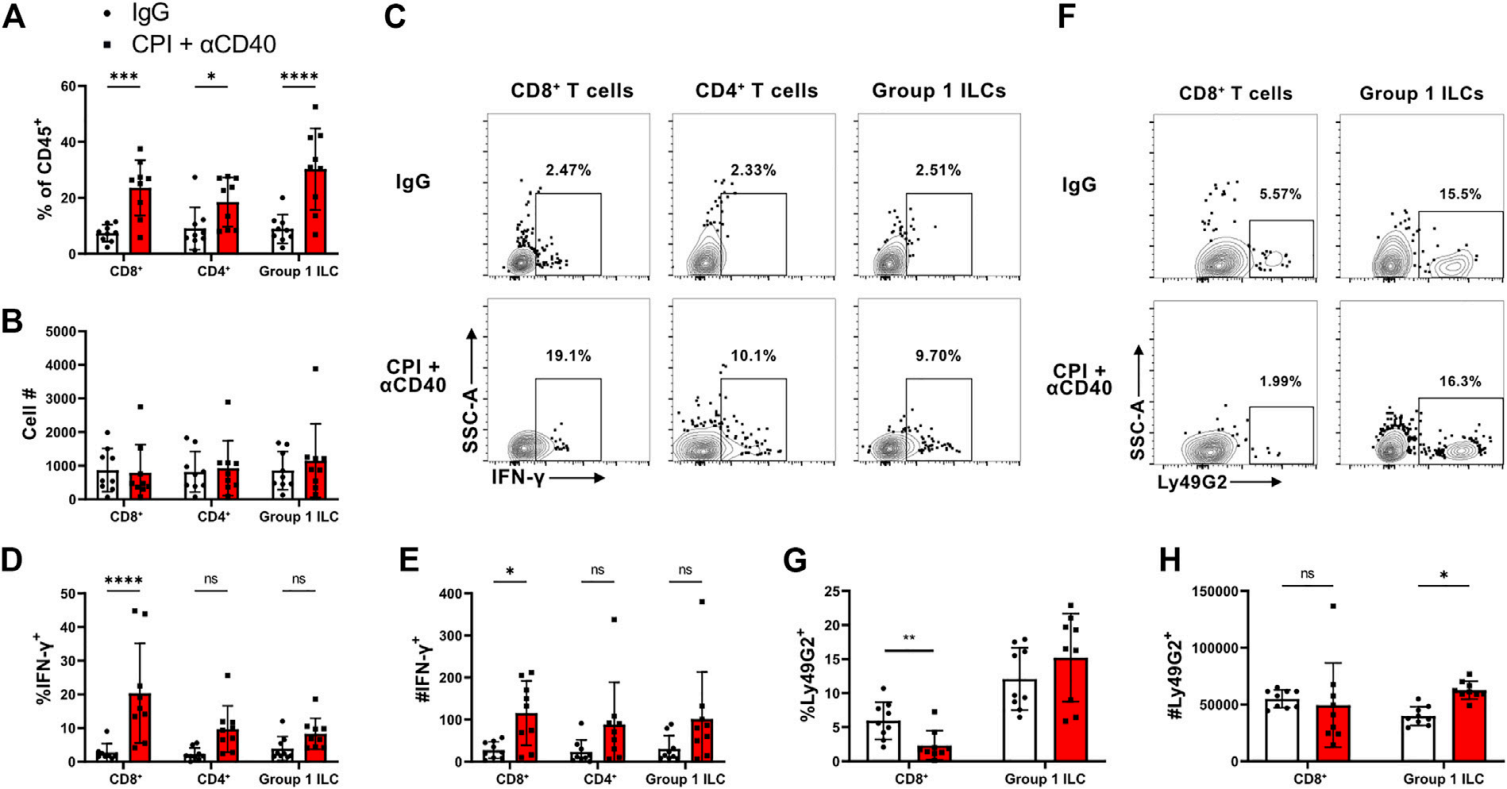
CPI40 treatment promotes maintenance and activation of TILs in the TME. Characterization of TILs 27* days post-implantation (*N* = 9 per group). Mice were injected i.p. with BFA 6 h prior to harvest. Figure shows the frequency and number of **(A, B)** TIL lineage, **(C–E)** IFN-γ producers, and **(F–H)** CD8^+^ T_reg_ cells. Holm-Šídák multiple comparison test was run in all cases. This experiment was performed once.

CPI40 has the potential to expand a T cell response within the tumor TME. Thus, we characterized immune cell infiltrate 6 days after CPI40 treatment *via* flow cytometry to understand alterations in lymphocyte representation and function. We initially confirmed that CD4^+^ and CD8^+^ T cells and innate lymphoid (ILC) cells were increased in frequency (Figure 2A). Interestingly, there was no indication of an increase in lymphocyte numbers, suggesting that other hematopoietic cells were reduced within the TME as a function of treatment (Figure 2B).

IFN-γ is a hallmark cytokine released by effector tumor infiltrating lymphocytes(TIL) populations. We assessed native TIL cytokine production within the TME by injecting animals with brefeldin A 6 h prior to sacrifice (Figures 2C–E). Although we found that CD4^+^ and CD8^+^ T cells and ILCs skewed towards enhanced IFN-γ production in the CPI40 treated group, the most robust phenotype was associated with CD8^+^ T cells. This suggests that CPI40 has the potential to enable CD8^+^ T cells to elicit anti-tumor functions within the TME.

Studies have indicated that CD4^+^ T_reg_ cells are diminished within the TME of mice treated with α-CTLA4 and α-CD40 in the context of PDAC. (Morrison et al., 2020). NK cell-associated MHC I receptors have been shown to be expressed by CD8^+^ T_reg_ cell populations (Kim et al., 2011). However, CD8^+^ T_reg_ cells have never been characterized in the context of tumor immunity. Ly49G2 is an MHC I receptor commonly expressed on NK cells and has been shown to be expressed on a discreet population of CD8^+^ T_reg_ cells (Kim et al., 2011). As expected, Ly49G2 can be found within the ILC compartment in the TME (Figures 2F–H). Interestingly, there were fewer Ly49G2^+^ CD8^+^ T cells in the CPI40 treated group, suggesting there is a higher CD8^+^ effector to CD8^+^ T_reg_ cell ratio in CPI40 treated mice. These data suggest that CPI40 treatment alters the CD8^+^ effector to CD8^+^ T_reg_ ratio.

We repeated the experiment in an orthotopic setting to verify that our initial findings would hold true in this context. CPI40 treatment had a robust effect on orthotopically implanted tumor outgrowth, as had been seen in subcutaneously implanted tumors (Supplementary Figures S1A, B). Additionally, we observed a similar increase in TIL frequency in the TME (Supplementary Figures S2). Despite similar numbers of total TILs (Supplementary Figures S2) there is clearly an increase in the density of CD8^+^ effector cells when normalizing cell number to the mass of the tumor (Supplementary Figures S2 **)**. These data suggest that CD8^+^ T cells are maintained during control of tumor outgrowth.

The presence of T_reg_ cells was analyzed after treatment with CPI40 in an orthotopic setting. Results showed a reduced representation of both conventional CD4^+^ CD25^+^ T_reg_ cells and CD8^+^ Ly49G2^+^ T_reg_ cells in mice treated with CPI40. (Supplementary Figures S2). Again, this suggests that CPI40 therapy polarizes the TME immune landscape to be comprised of more effector, rather than suppressive, constituent cell populations.

### Regulation of PD1 expression in response to CPI40

PD-1 is a well-known inhibitory receptor linked to both lymphoid cell activation and exhaustion. The expression level of PD-1 on a per cell basis correlates with the activation state of a cell, with higher expression associated with acute activation and lower expression associated with exhaustion (Wherry and Kurachi, 2015). To determine which cells are responding to PD-1 blockade and how they respond to CPI40, PD-1 expression was analyzed on TIL populations. (Supplementary Figures S2). CPI40 therapy led to no change in the proportion of PD-1^+^ CD8^+^ T cells, but there was a sharp decrease in expression on a per-cell basis. Additionally, there was a higher representation of PD-1^+^ CD4^+^ T cells, with no change in expression on a per-cell basis. These findings suggest that CD8^+^ T cell expression may reflect acute activation rather than exhaustion during CPI40 treatment, whereas CD4^+^ T cells have a higher frequency of PD-1 expression, which could potentially limit their effector potential in the absence of PD-1 blockade.

### CPI40 induces CD4^+^ T cell-dependent immune memory to PDAC

**FIGURE 3:**
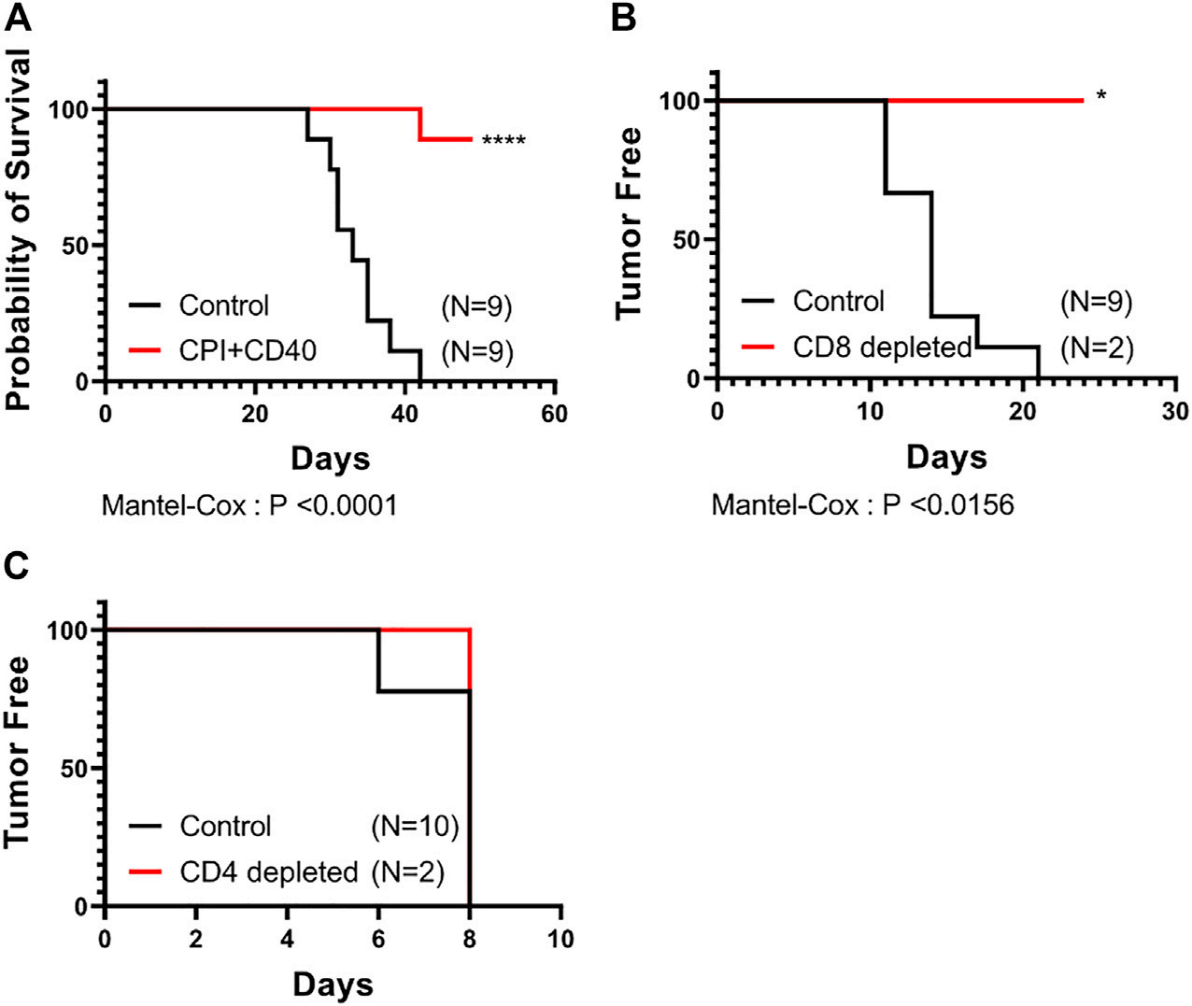
CPI40 treatment leads to prolonged survival that is dependent upon CD4^+^ T cells. **(A)** Survival curves of treated groups (N = 9 per group). Tumor free survival after depletion of **(B)** CD8^+^ then **(C)** CD4^+^ T cells in naïve or CPI40 survivors. Depletions were performed sequentially in two independent experiments with the same subjects from the CPI40 treated group. Mantel-Cox analysis was performed in all cases. These experiments were performed once.

Our subcutaneous CPI40 experiment (Figure 1) resulted in 6 of 8 CPI40 survivors that fully controlled tumors (no palpable mass) (Figure 3A). Two of 6 of these mice redeveloped tumors naturally 15- and 28-day post observations of no palpable mass (i.e., transiently dormant tumor cells were still present). We investigated whether CPI40 treatment induced immune memory and rechallenged the remaining mice with KPC4662 on their opposing flank. 2/4 mice showed no signs of tumor development after rechallenge 36 days post-implantation (data not shown), suggesting that CPI40 treatment can induce tumor-specific memory. We then tested whether adaptive immunity was dependent upon CD8^+^ T cells by depleting the remaining mice with CD8^+^ T cell depleting antibody. Both mice were able to reject the tertiary implanted tumors (Figure 3B). We then tested whether CD4^+^ T cells were involved using a similar approach. Interestingly, both mice developed tumors after a fourth rechallenge (Figure 3C). These data suggest that CD4^+^ T cells are necessary for CPI40-induced adaptive immunity against KPC4662 tumor cells.

### Circulating lymphoid cells are dispensable for PDAC growth control

We have previously shown that αCD40-mediated, T cell dependent tumor control does not require the recruitment of newly primed effector T cells in well-infiltrated melanomas (Stevens and Bullock, 2021). However, PDACs are reported as being immunologically “cold” due to a paucity of TILs with limited effector activity in the tumor microenvironment. This suggests that CPI40’s efficacy may depend on freshly primed T cells. We therefore tested whether a *de novo* T cell response is responsible for CPI40-driven control of pancreatic tumors. FTY720 is a drug that activates S1P1 receptor which induces its downregulation and subsequently prevents T cell egress from lymphoid tissues. FTY720 had no impact on tumor control in the context of CPI40 (Supplementary Figures S3A, B). These data suggest that a *de novo* induced response, driven by T cells that emigrate from the draining lymph node, is unnecessary for tumor control and that the cells intrinsic to the tumor at time of therapy are sufficient for the observed activity of the combination therapy.

### Role of the CD27:CD70 axis in CD40-dependent growth control

**FIGURE 4:**
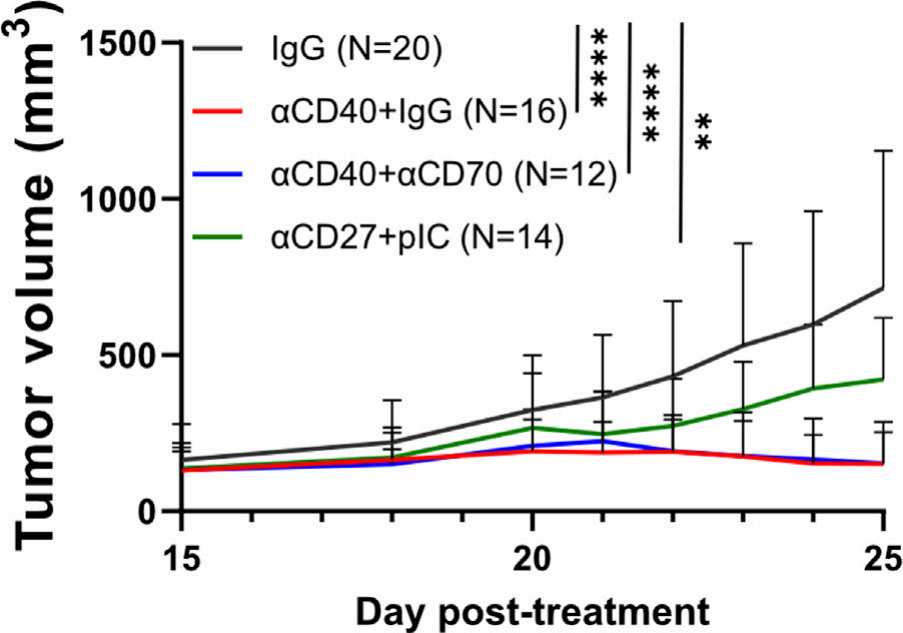
Interrogating the role of the CD27:CD70 axis in CD40-mediated tumor control. 250k KPC-7940b tumor cells were implanted s.c into C57BL/6 mice. Mice were treated with clg (600 ug) (D-1, D2, D5), CD70-blockade (600 ug) (D-1,D2, D5 (*N* = 4), or D-2 and D3 (*N* = 8)), CD40-agonist (100 ug) (D0), or CD27-agonist (100 ug) and pIC (100 ug) (D0, D3, D9). Tumor volumes over time are shown for each treated group. Standard deviations are shown for each time point. Mixed-effects analysis was performed, *p* < 0.0001 across time and treatment. These are aggregate data from two independent experiments.

Our previous studies have indicated an ability for CD40 stimulation along with pIC to enhance T cell responses within melanomas without the need for checkpoint blockade (Stevens and Bullock, 2021). Further, we have demonstrated that a major component of the efficacy of CD40 stimulation occurs *via* the induction of CD70 on cDCs (Bullock and Yagita, 2005). Others have demonstrated that the CD70:CD27 axis potentiates tumor specific T cell expansion after CD40 stimulation (Oba et al., 2020). Thus, we also hypothesized that blockade of CD70 would abrogate CD40-mediated control of subcutaneous PDAC tumors. Accordingly, we treated mice with just CD40 agonist mAb with, or without, a CD70 antagonist mAb. As we have previously demonstrated that the combination of CD27 (the receptor for CD70) and IFNαβR stimulation can substitute for CD40 stimulation, we reasoned that circumventing CD70-signaling with a CD27-agonist mAb would promote tumor control. Therefore, we also included a group that received plC and an agonistic CD27 mAb. Mice that were given αCD40 exhibited robust tumor control, regardless of blocking CD70 (Figure 4). While the degree of tumor control induced by the combination of plC and αCD27 is significant, it does not reach a comparable level to that provided by αCD40 treatment. These results indicate that CD40-specific stimulation is required for optimal PDAC tumor control, independent of its downstream signaling through CD70.

**FIGURE 5:**
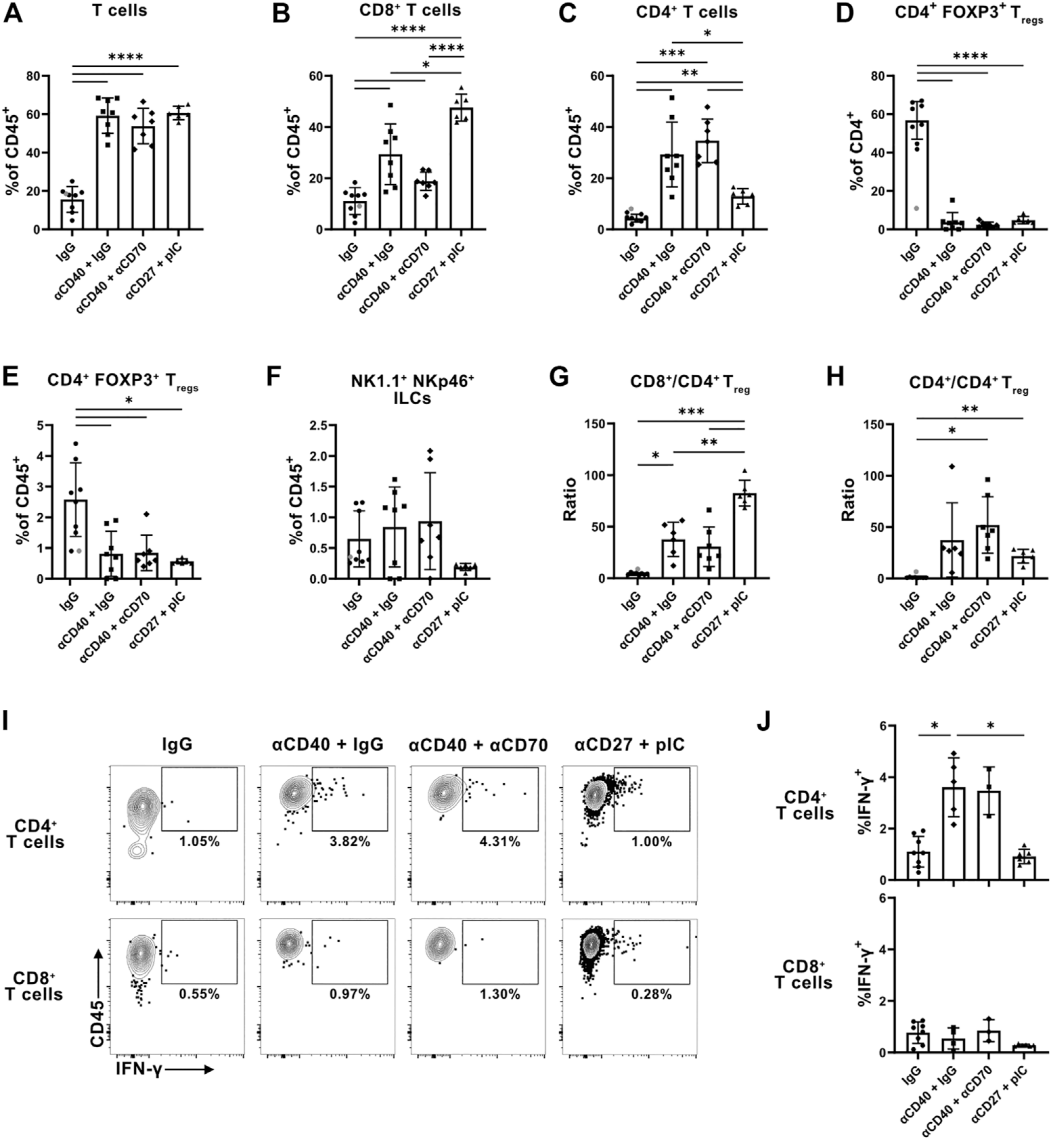
**Stimulation of CD40 improves representation and cytokine production of CD4** ^+^ **T cells within tumors independent of CD70-signaling.** Evaluation of T cell infiltration into KPC-7940b tumors 23 days post implantation. **(A)** Frequency of total T cells among CD45^+^ immune cells in the TME. **(B)** Frequency of CD8^+^ and **(C)** CD4^+^ T cells among all CD45^+^ immune cells. **(D)** Frequency of FOXP3^+^ T_reg_ cells among CD4^+^ T cells and **(E)** total CD45^+^ immune cells. **(F)** Frequency of NK1.1^+^ NKp46^+^ ILCs among CD45^+^ immune cells. **(G)** Ratio of CD8^+^ **(H)** and CD4^+^ T cells to CD4^+^ FOXP3^+^ T_reg_ cells. **(I)** Representative flow plots of IFN-γ production by CD4^+^ and CD8^+^ T cells. **(J)** Frequency of IFN-γ^+^ events among CD4^+^ and CD8^+^ T cells. Samples with fewer than 100 CD4^+^ or CD8^+^ T cells were insufficient for analysis of IFN-γ production and subsequently excluded. Grey dot denotes statistically determined outlier which was excluded from the analysis. Brown-Forsythe and Welch ANOVA tests were performed in all cases. This experiment was performed once.

After initiation of immunotherapeutic treatments, we measured tumors daily until there was either clear tumor control or a lack thereof. Upon reaching this point, we characterized the immune cell infiltration of tumors *via* flow cytometry to evaluate immunological changes in the TME. Changes in the numbers of specific cell types were not informative regarding comparison between groups due to the small size of immune populations present in resolved tumors (data not shown). However, the tumors of mice that received treatments display a three-fold increase in the frequency of T cells present, indicating that the various immunotherapies can mobilize a T cell response (Figure 5A). CD8^+^ T cells are often considered vital mediators of the antitumor response, however, the αCD27 and pIC treated mice exhibit suboptimal tumor control, despite having the highest representation of CD8^+^ T cells (Figure 5B). On the contrary, the frequency of CD4^+^ T cells is highest among the well-controlled αCD40 treated tumors, even when CD70-signaling is blocked (Figure 5C). However, CPI40 does not induce the same pattern, perhaps due to a CPI specific mechanism (Figure 2A). The proportion of CD4^+^ T_reg_ cells among CD4^+^ T cells and total immune cells declined similarly among all treated groups, suggesting that differences in the degree of tumor control may be due to the accumulation of CD4^+^ T cells (Figures 5D, E). This does not extend to the ILC compartment, as the frequency of NK1.1 + NKp46 + ILCs does not change after αCD40 monotherapy, despite an increase after CPI40 (Figure 5F).

Considered a benchmark for measuring the antitumor immune response, the ratio of CD8^+^ T cells to T_reg_ cells only modestly increased in the tumors of αCD40 treated mice but increased more significantly in the pIC and αCD27 treated tumors (Figure 5G). Conversely, the ratio of CD4^+^ T cells to CD4^+^ T_reg_ cells trended towards being the highest among the αCD40 groups and moderate in the pIC and αCD27 group (Figure 5H). This further supports a role for CD40 stimulation in skewing T cell infiltration of tumors towards CD4^+^ T cells, even in the absence of signaling through CD70.

Beyond examining αCD40-specific changes in tumor infiltration by T cells, we wanted to determine if there are αCD40 dependent changes in T cell activity. To assess *in vivo* IFN-γ production, we injected mice with brefeldin A 6 hours before sacrificing. This yielded evidence of improved IFN-γ production by CD4^+^ but not CD8^+^ T cells, even in mice treated with the αCD70 antagonist. (Figures 5I, J). pIC and αCD27 treatment did not change IFN-γ production by CD4^+^ T cells (Figures 5I, J). Thus, the enhancement of CD4^+^ T cell function requires CD40 stimulation but occurs independent of CD70-signaling. Immunotherapeutic treatments did not alter CD8^+^ T cell production of IFN-γ, further suggesting that a CD4^+^ T cell effector signature specifically coincides with robust tumor control (Figure. 6A and C).

## Discussion

In this study, we have provided further evidence that targeting the stimulation of CD40, with or without concomitant use of immune checkpoint inhibition, is a potent mechanism for improving pancreatic cancer control, both in orthotopic and subcutaneous settings. The combination therapy promotes the presence of activated T cell effector populations with increased cytokine production, and this therapy remains effective when T cells are prevented from leaving the draining lymph nodes. We further find that the combination therapy results in substantial immunological protective memory that is remarkably dependent of CD4^+^ T cells, not CD8^+^ T cells. Finally, the efficacy of CD40 stimulation appears independent of its ability to induce the expression of CD70, though targeting CD27 and IFNαβR can modestly replicate the efficacy of CD40 agonism.

In previous studies we determined that CD40-mediated stimulation of melanoma intratumoral T cells is sufficient to promote tumor control without further contribution of T cells primed in draining lymph nodes (Stevens and Bullock, 2021). However, in that study, we needed to provide antigen to achieve consistent tumor control. In this study of pancreatic cancer, we find CD40 stimulation is sufficient, without further need to provide antigen. This suggests that the makeup of CD40-expressing cells and their ability to either present already acquired antigen or respond with direct tumoricidal activity to release more antigen is distinct in different models of cancer. Further, in the melanoma model control was transient, and the activation of T cells rapidly diminished. Conversely, only a single infusion of αCD40 was necessary to drive durable tumor control in this current study. Thus, resistance mechanisms that arise after CD40 stimulation in these models appear to be different. This becomes particularly relevant when considering the inconsistent outcomes for the PRINCE combination therapy clinical trial, in which only a subset of pancreatic cancer patients received a benefit (Padrón et al., 2022). Understanding how well mouse models correlate with the human experience, and whether they can reveal distinct resistance mechanisms, will be imperative for designing next-generation trials with increased efficacy.

Consistent with previous studies, we find that resolution of tumors after combination therapy provides potent resistance to rechallenge, demonstrating the establishment of memory against pancreatic tumor antigens. While initial control of these tumors depended upon either CD4^+^ or CD8^+^ T cells, rejection by memory T cells was attributed to the CD4^+^ T cell subset. Given that the mice were challenged at distal sites, this argues that central, rather than tissue-resident, memory CD4^+^ T cells are responsible for this protection. This outcome suggests that the CD4^+^ T cell responses within patients should be examined as a potential biomarker of efficacy, and that strategies that promote CD4^+^ T cell immunity may be particularly effective in pancreatic cancer. Due to our prior experience with CD40-mediated vaccination, we directly tested the hypothesis that the effectiveness of CD40 agonists was due to their capacity to drive CD70 expression, with the resultant engagement of CD27 on effector T cell populations. Counter to our hypothesis, we find that tumor control and T cell presence and activation in the TME are independent of CD70, consistent with a recent study genetically showing that CD70 is dispensable for the activation of T cells by DC in a tumor setting (Wu et al., 2022).

To some degree, targeting CD27 along with IFNαβR, which we have previously shown to be a potent driver of CD8^+^ T cell responses to vaccination (Dong et al., 2012; Dong et al., 2019) and to promote tumor control (Roberts et al., 2010; van de Ven and Borst, 2015; Riccione et al., 2018), had limited success in this model of pancreatic cancer. This held true, even though αCD27+pIC led to a large expansion of CD8^+^ T cells within the pancreatic TME and a reduction in T_reg_ cells. Notably, αCD27 and pIC did not improve the proportion of T cells producing IFN-γ compared to that achieved by αCD40. This suggests that there are suppressive mechanisms in the TME that are subverted by CD40 but not CD27 stimulation (which likely directly acts on intratumoral T cells). Immature myeloid cells are an obvious candidate, and future studies that are intended to identify the therapeutic target of CD40 stimulation will help tease apart this distinction. Further, given the T cell dependency of the tumor control achieved with CD40 stimulation, we conclude that CD40 stimulation is promoting T cell responses by a pathway independent of CD70. CD40 stimulation can promote a variety of costimulatory molecules on cDC, including 4-1BB and OX40, and cytokines such as IL-12 and Type-I interferons, each of which could provide the link to expanded T cell function. It is of special interest to note that OX40 (CD134) stimulation has been shown to strongly promote CD4^+^ T cell responses (Croft, 2010; Kurche et al., 2010). Future studies will examine the contribution of these molecules with respect to the tumor control achieved by CD40 stimulation.

It is of note that we observe that the vast majority of the efficacy of the combination therapy is driven by CD40 stimulation, and that CPI therapy on its own was ineffective (data not shown). Studies from other labs have argued that increased tumor control is achieved by the inclusion of CPI (Rech et al., 2018; Morrison et al., 2020)) Reasons for this difference are not readily apparent, but the nature of the immune cell infiltration and myeloid cell makeup can differ considerably between institutions, often as a function of different microbiomes. This possibility is currently being studied.

## Data Availability

The raw data supporting the conclusion of this article will be made available by the authors, without undue reservation.
