# Supplementary material for: Interrogating the CD27:CD70 axis in αCD40-dependent control of pancreatic adenocarcinoma
Source: Front Cell Dev Biol. 2023 Apr 12;11:1173686. doi: 10.3389/fcell.2023.1173686 (PMC10130518; doi:10.3389/fcell.2023.1173686)
Supplement: Supplementary file 1 [file DataSheet1.pdf]

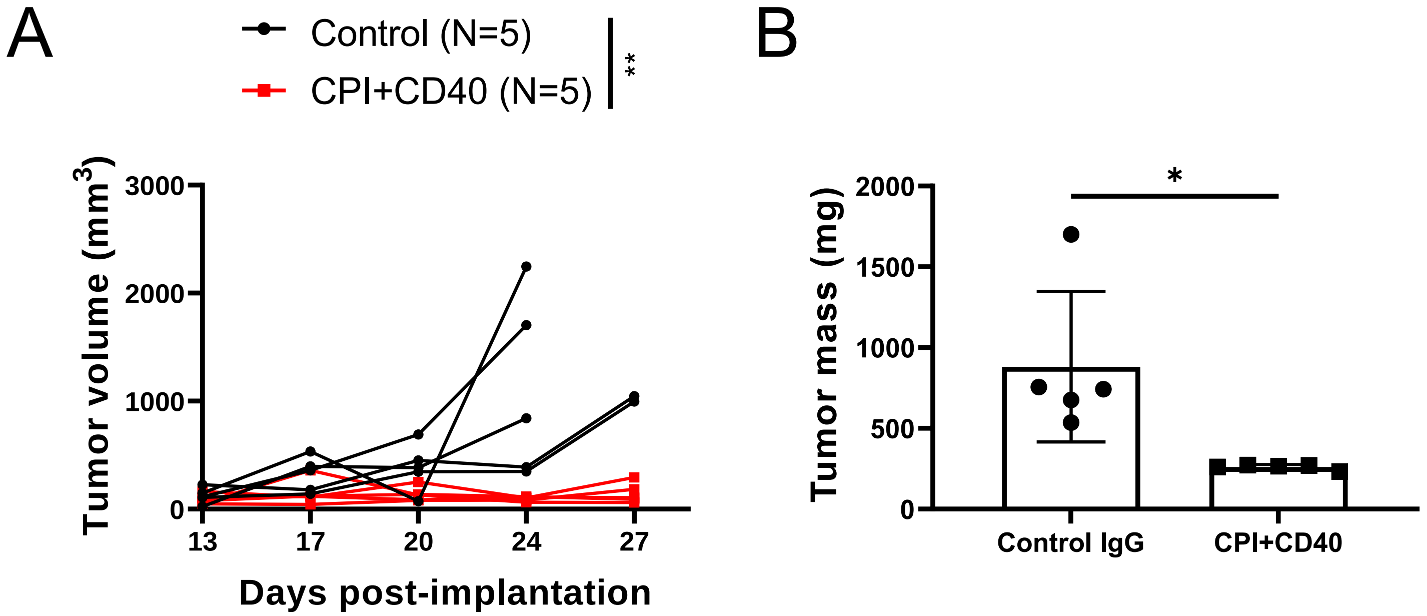

Supplemental Figure 1. CPI40 treatment controls tumor outgrowth in an orthotopic setting. 500k KPC4662 cells in a 1:1 Matrigel to RPMI mixture were implanted, via surgery, into the tail of the pancreas. Mice were treated on days 10, 13, 16, and 19 with αPD1 (200ug) and on day 13 with αPD1 (200ug) and αCD40 (100ug). Tumor volume was then determined via ultrasonography. **(A)** tumor volume measurements for individual mice from the control IgG and CPI40 treated groups (N=8 per group). Mixed-effects analysis was performed, P <0.0001 across time and treatment. **(B)** Tumor mass of mice sacrificed at day 20 (N=5 per group). Student's T-test was performed. This experiment was performed once.

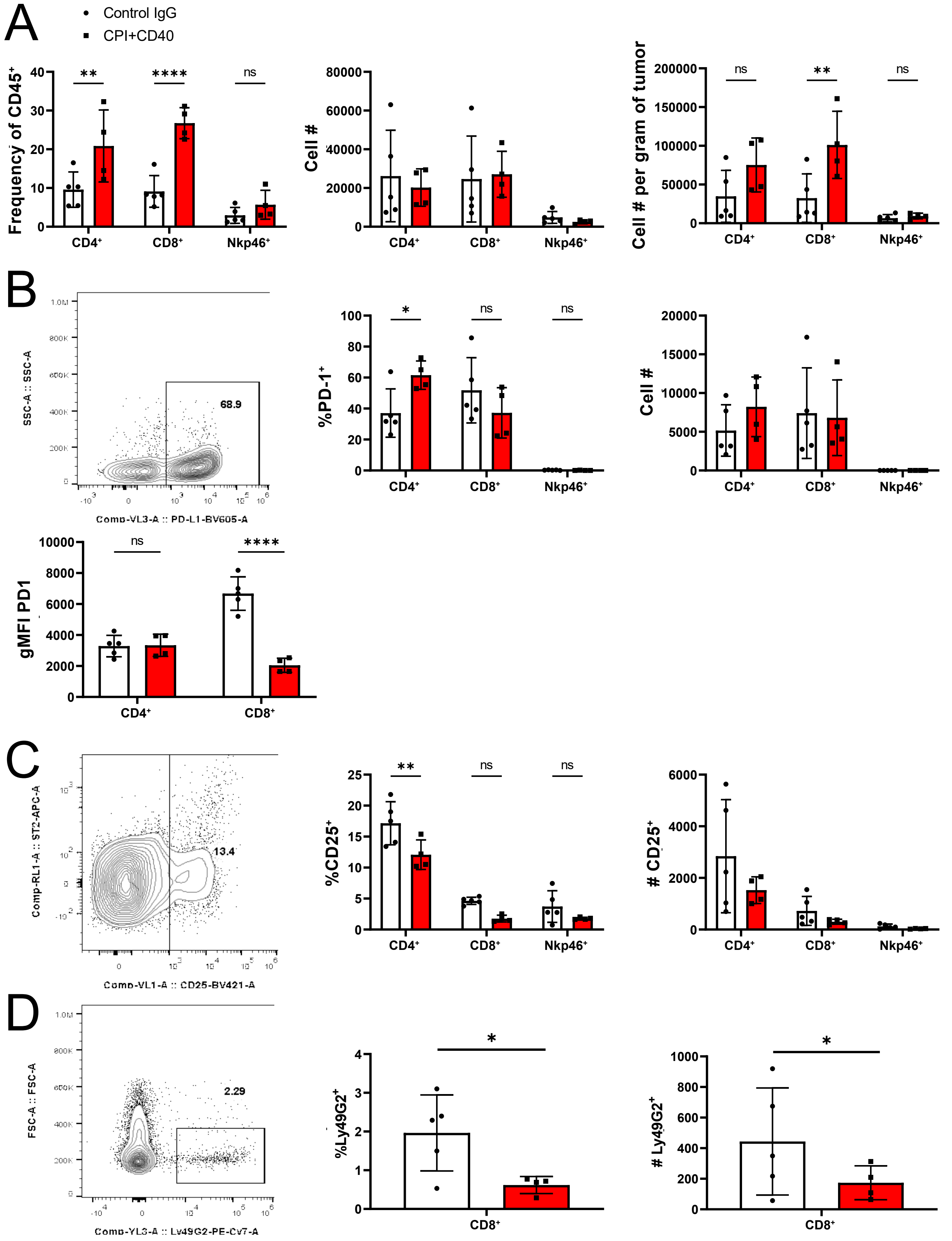

Supplemental Figure 2. CPI40 treatment promotes maintenance and activation of TILs in the orthotopic TME.

Characterization of TILs 27 days post-implantation (N=9 per group). Mice were injected i.p. with BFA 6hr prior to harvest. **(A)** TIL lineage frequency, **(B)** PD1 expression, **(C)** CD4⁺ T_{reg} and **(D)** CD8⁺ T_{reg} cell frequency. Holm-Šídák or Student's T test were performed where applicable. This experiment was performed once.

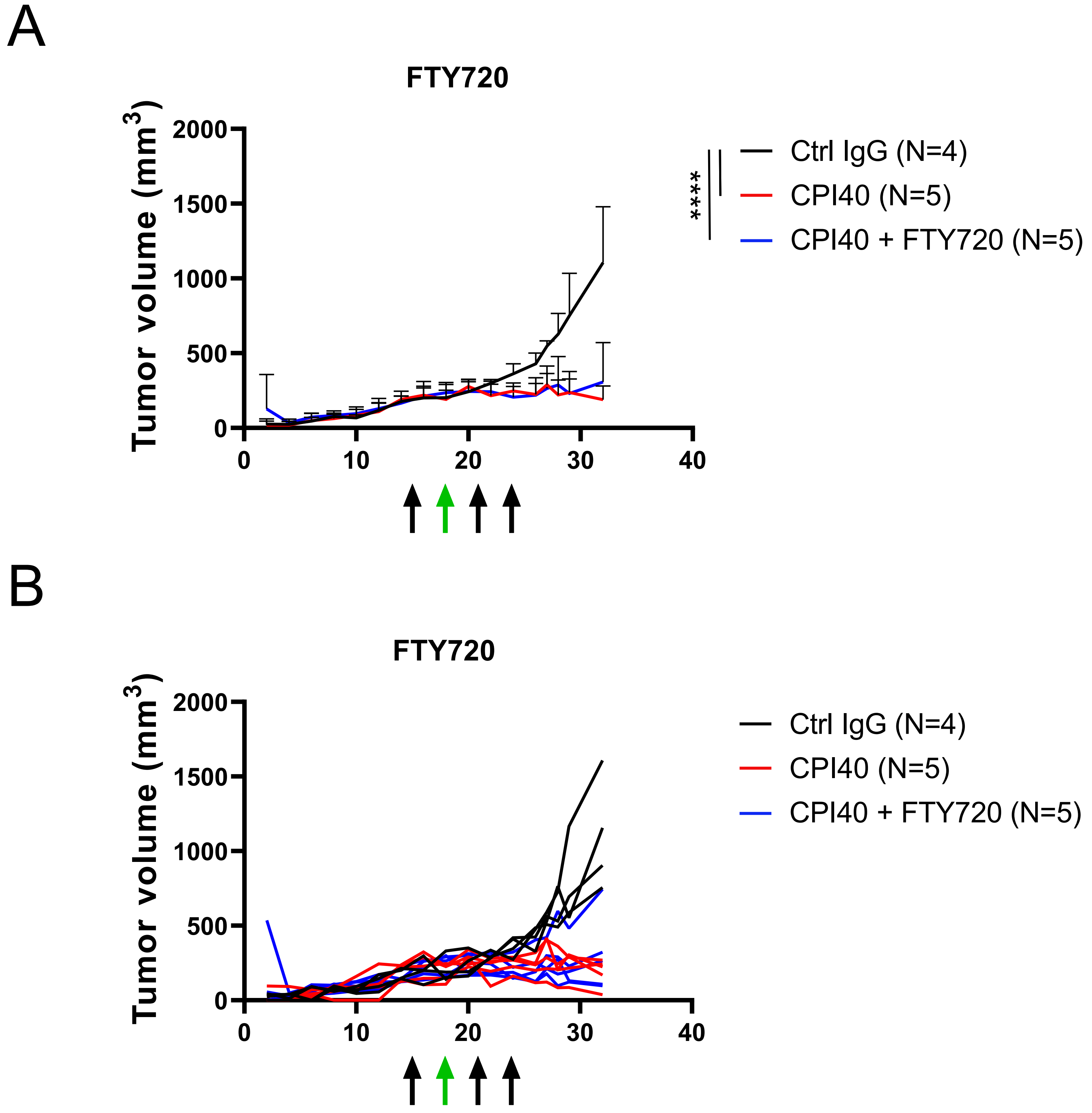

Supplemental Figure 3. Interrogating the role of the circulating lymphocytes in CPI40-mediated tumor control.

2.5 x 10^5 KPC-4662 tumor cells were subcutaneously implanted into C57BL/6 mice supplied from The Jackson Laboratory. **(A)** Average and **(B)** individual tumor volumes over time for each treated group. Standard deviations are shown for each time point. Mice were i.p. injected with cIg (200ug), FTY720 (25ug), CD40-agonist (100ug), PD1 (200ug), and CTLA4 (200ug) on the indicated days. Mixed-effects analysis was performed, P <0.0001 across time and treatment. This experiment was performed once.

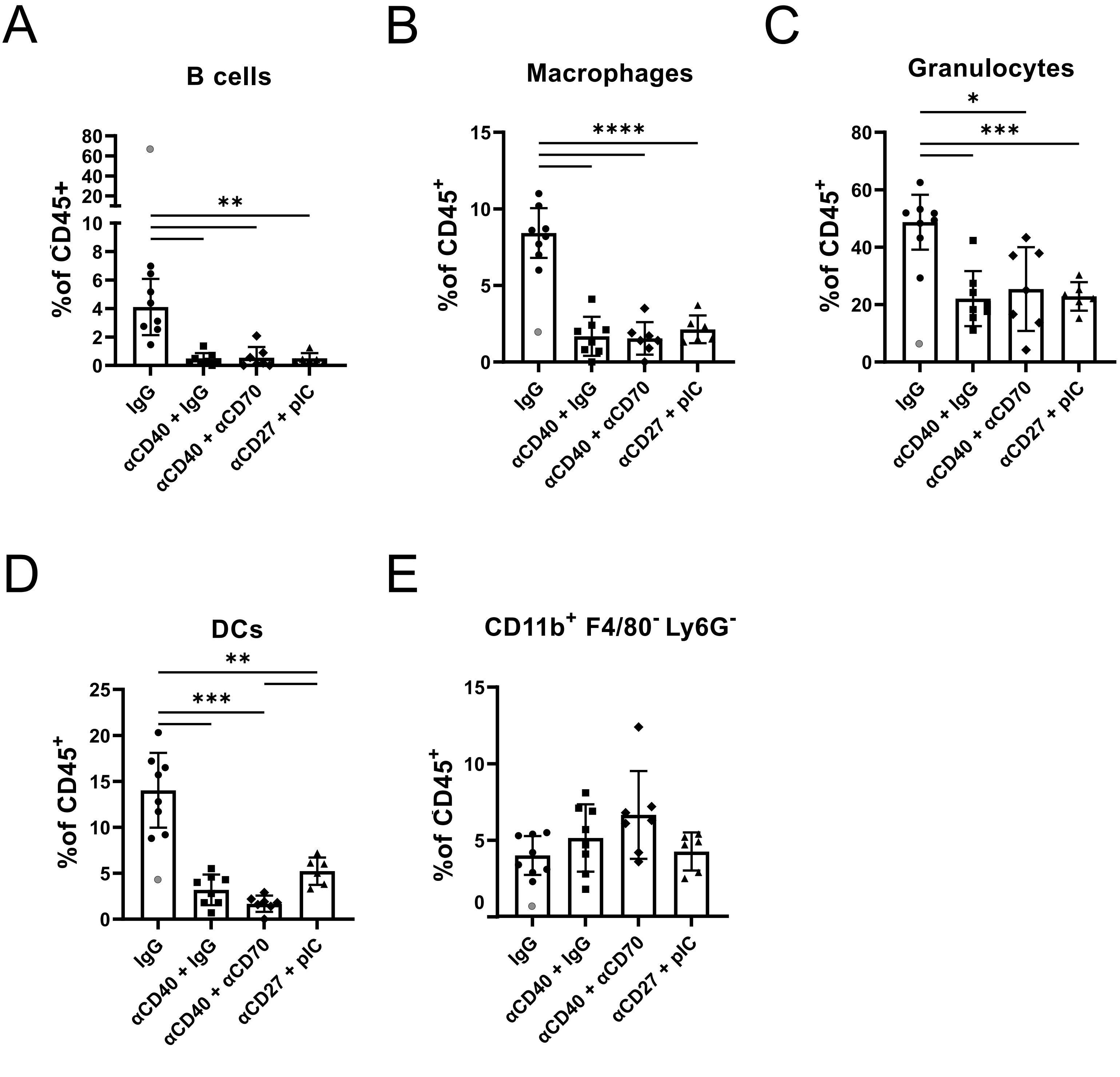

Supplemental Figure 4. Immunotherapeutic treatments reduce the frequencies of myeloid cells within tumors.

Evaluation of immune subsets present in KPC-7940b tumor cells 23 days post-implantation. Frequency of **(A)** $CD19^+$ B cells, **(B)** $F4/80^+$ $CD11b^+$ macrophages, **(C)** $Ly6G^+$ $CD11b^+$ granulocytes, **(D)** $CD11c^+$ $MHCII^+$ DCs, and **(E)** $CD11b^+$ $F4/80^-$ $Ly6G^-$ myeloid cells. Grey dot denotes statistically determined outlier which was excluded from the analysis. Forsythe and Welch ANOVA tests were performed in all cases. This experiment was performed once.
